# A Novel Trans-Tracheostomal Retrograde Inhalation Technique Increases Subglottic Drug Deposition Compared to Traditional Trans-Oral Inhalation

**DOI:** 10.3390/pharmaceutics15030903

**Published:** 2023-03-10

**Authors:** Raviv Allon, Saurabh Bhardwaj, Josué Sznitman, Hagit Shoffel-Havakuk, Sapir Pinhas, Elchanan Zloczower, Yael Shapira-Galitz, Yonatan Lahav

**Affiliations:** 1 Department of Otolaryngology, Head and Neck Surgery, Kaplan Medical Center, Rehovot 76100, Israel; 2 Faculty of Medicine, Hebrew University of Jerusalem, Rehovot 76100, Israel; 3 Department of Biomedical Engineering, Technion—Israel Institute of Technology, Haifa 3200003, Israel; 4 Department of Otolaryngology, Head and Neck Surgery, Rabin Medical Center, Petach-Tikva 4941492, Israel; 5 Sackler Faculty of Medicine, Tel Aviv University, Tel Aviv 6997801, Israel

**Keywords:** laryngotracheal stenosis, subglottic stenosis, computational fluid dynamics, airflow, particle transport, inhaled corticosteroids, metered dose inhalers

## Abstract

Subglottic stenosis represents a challenging clinical condition in otolaryngology. Although patients often experience improvement following endoscopic surgery, recurrence rates remain high. Pursuing measures to maintain surgical results and prevent recurrence is thus necessary. Steroids therapy is considered effective in preventing restenosis. Currently, however, the ability of trans-oral steroid inhalation to reach and affect the stenotic subglottic area in a tracheotomized patient is largely negligible. In the present study, we describe a novel trans-tracheostomal retrograde inhalation technique to increase corticosteroid deposition in the subglottic area. We detail our preliminary clinical outcomes in four patients treated with trans-tracheostomal corticosteroid inhalation via a metered dose inhaler (MDI) following surgery. Concurrently, we leverage computational fluid-particle dynamics (CFPD) simulations in an extra-thoracic 3D airway model to gain insight on possible advantages of such a technique over traditional trans-oral inhalation in augmenting aerosol deposition in the stenotic subglottic region. Our numerical simulations show that for an arbitrary inhaled dose (aerosols spanning 1–12 µm), the deposition (mass) fraction in the subglottis is over 30 times higher in the retrograde trans-tracheostomal technique compared to the trans-oral inhalation technique (3.63% vs. 0.11%). Importantly, while a major portion of inhaled aerosols (66.43%) in the trans-oral inhalation maneuver are transported distally past the trachea, the vast majority of aerosols (85.10%) exit through the mouth during trans-tracheostomal inhalation, thereby avoiding undesired deposition in the broader lungs. Overall, the proposed trans-tracheostomal retrograde inhalation technique increases aerosol deposition rates in the subglottis with minor lower-airway deposition compared to the trans-oral inhalation technique. This novel technique could play an important role in preventing restenosis of the subglottis.

## 1. Introduction

Stenosis of the subglottis and upper trachea represents one of the most challenging problems to treat in the field of otolaryngology. Although patients often experience improvement after surgical intervention, recurrence rates remain high at up to 80% of patients and can occur within days to months following surgery, leading to further multiple and various interventions [1,2,3,4,5,6]. A pursuit for measures to maintain surgical results and prevent recurrence is ongoing. Restenosis occurs due to the inflammatory reaction and fibrosis of the tubular organ that continue after the surgery, creating contraction in a centripetal vector that collapses the airway and decreases the diameter of the affected segment [7].

Due to their antifibrotic and anti-inflammatory properties [8], corticosteroids are often used in the management of subglottic and tracheal stenosis. Administration routes include systemic (oral or intravenous) [9], intralesional injection under general [2,5,10] or local [11,12,13,14] anesthesia, and finally topically, as orally inhaled corticosteroids. The last option is especially appealing, as it allows easy, at-home use that can potentially extend the intervals between surgeries or in-office procedures and does not bear the adverse effects of prolonged systemic steroid treatment. Inhaled corticosteroids are often prescribed, and some case series support its efficacy in treating airway stenosis [15,16,17].

However, in our own clinical experience, the ability of inhaled corticosteroids to affect the stenotic subglottic region using oral or nasal inhalation is negligible, especially in the tracheotomized patient, as the drug seldom reaches and affects the stenotic region in this technique. In the only randomized controlled study published to date, the use of inhaled corticosteroids in laryngotracheal stenosis following balloon dilation showed no benefit [18]. Notably, such observations were supported by computational fluid-particle dynamics (CFPD) simulations that showed that only 2.6% of the orally inhaled drug particles effectively deposited in the subglottic region [19].

Patients with tracheal and subglottic stenosis are often tracheostomy-dependent. As such, the patient breathes via a tracheostomy cannula and speaks by capping the cannula and exhaling the air, either around the cannula or through an upper fenestration in the cannula aiming towards the vocal folds via the subglottic area. Here, we hypothesize that if inhaled corticosteroids are given via a fenestrated tracheostomy tube during air exhalation, the proximity of the fenestration to the subglottis would allow for significant drug concentration to initially reach to the subglottis and thus increase the amount of drug deposited in the stenotic subglottic area, unlike traditional trans-oral inhalation of the drug.

In the present study, we detail our recently developed trans-tracheostomal, retrograde, corticosteroid inhalation (TRCI) technique in an effort to achieve higher drug concentration in the subglottic area while minimizing superfluous aerosol deposition in the broader lungs. We detail our preliminary clinical outcomes in four patients treated by TRCI via a metered dose inhaler (MDI) following airway surgery. Concurrently, we used computational fluid-particle dynamics (CFPD) simulations in an extra-thoracic 3D airway model to compare our novel TRCI with a traditional trans-oral inhalation technique in relation to local subglottic aerosol deposition for the treatment of subglottic stenosis.

## 2. Materials and Methods

### 2.1. Case Series

The study was approved by the ethics committee of Kaplan Medical Center, Rehovot, Israel. Patient data were extracted from the institute’s electronic medical records (EMRs) (Chameleon, Elad Software Ltd., Tel Aviv, Israel). We included all patients from our institution (Kaplan Medical Center, Rehovot, Israel) who had subglottic stenosis treated with trans-tracheostomal, retrograde, corticosteroid inhalation from 2020 to 2022. All cases are reported according to the CARE case report guidelines [20].

### 2.2. Trans-Tracheostomal, Retrograde, Corticosteroid Inhalation (TRCI) Technique

**Figure 1:**
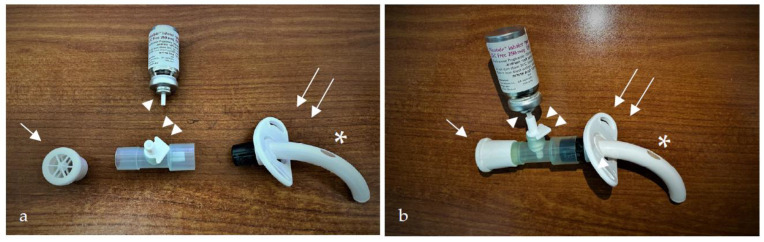
Photograph of the (**a**) disassembled and (**b**) assembled trans-tracheostomal retrograde device. The canister of the metered-dose inhaler (MDI) (arrowhead) is connected to a one-way T-shaped adaptor (two arrowheads). The adaptor is attached on its distal side to a one-way speaking valve (arrow) and on the proximal side to the fenestrated tracheostomy tube (two arrows). Notice the fenestration in the upper aspect of the cannula (asterisk).

Figure 1 presents the composition of the trans-tracheostomal retrograde device, which can be also viewed in the Supplementary Video S1. Briefly, the technique uses a commonly available, universal, T-shaped inhaler adaptor suitable for endotracheal and tracheostomy tubes. The adaptor is connected on its distal end to a one-way speaking valve and on the proximal other end to the fenestrated tracheostomy cannula. The “leg” of the T-shaped adaptor is connected to the inhaler canister with an arrowhead pointed to the direction of drug flow on activation. The speaking valve allows the patient to breathe in during the process, but prevents the escape of the air via the cannula’s orifice during the exhalation phase. The patient is guided to press the inhaler during air exhalation. With the speaking valve, all the aerosolized drug is forced upwards via the fenestration, towards the subglottic and vocal cords. When performed correctly, the patient and physician should observe some of the drug sprayed outwards through the mouth. (see Supplementary Video S2). Our treatment protocol uses a fluticasone propionate inhaler (i.e., MDI) with a dose of 250 micrograms per actuation, two puffs, administered three times daily (overall, 1500 micrograms daily).

### 2.3. Extra-Thoracic In Silico Airway Model

**Figure 2:**
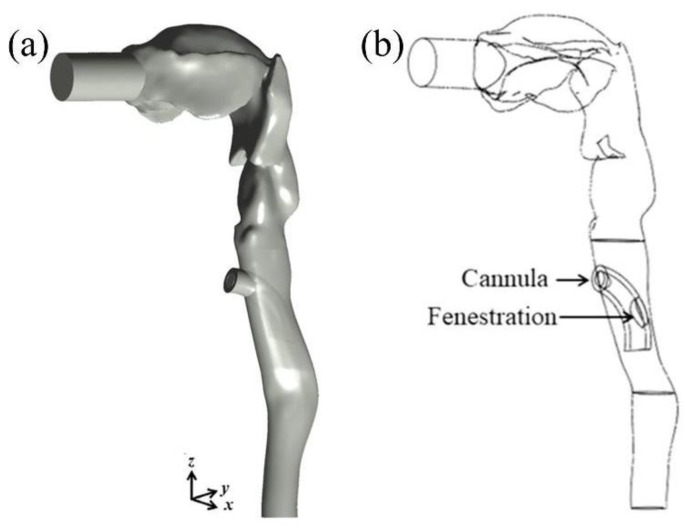
Computer-aided design (CAD) of the 3D extra-thoracic airway model introduced by Lizal et al. [21,22] and modified to include the simulation of a stoma and a cannula with fenestration. (**a**) General surface geometry and (**b**) wireframe view of the tracheostomy cannula and the corresponding fenestration at an estimated location of the 2nd tracheal ring shown in a sagittal plane.

For the CFPD simulations, we adopted the *SimInhale* benchmark model introduced by Lizal et al. [21,22] and assessed by Koullapis et al. [23]. We further modified the model to fit the tracheostomized patient by adding a stoma and tracheostomy cannula located at the level of the second tracheal ring. The measurements of a cuffless fenestrated Shiley^TM^ cannula size six were used (Shiley^TM^ 6CFN, Tracheostomy Tube Cufless with Inner Cannula, Fenestrated; Covidien^TM^, 15 Hampshire Street, Mansfield, MA, USA). The extra-thoracic airway model spans the mouth to the trachea, including the oral cavity, oropharyngeal, laryngeal and tracheal segments, as illustrated in Figure 2.

**Figure 3:**
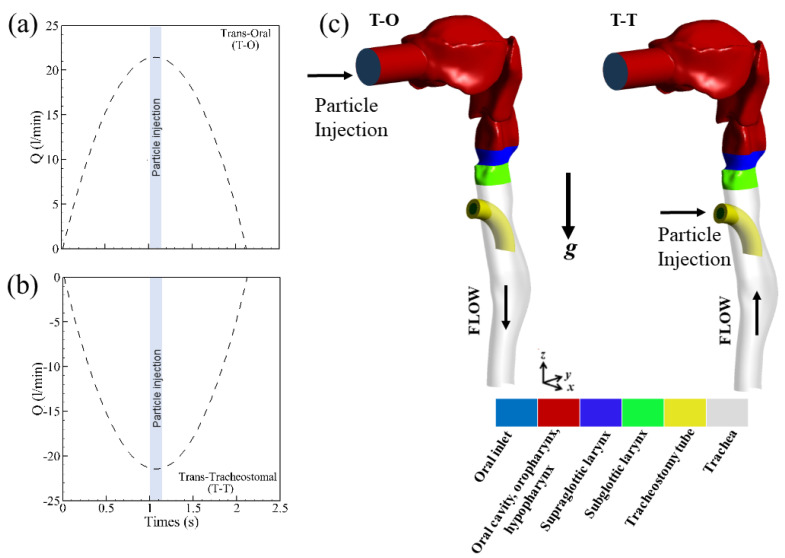
Modeled inhalation maneuver (i.e., flow rate over time) implemented for the (**a**) trans-oral and (**b**) trans-tracheostomal technique; shown in blue is the corresponding time window for particle injection (i.e., puff from MDI). Note that during (**a**) the trans-oral protocol, the patient inhales (i.e., positive airflow entering the mouth of the patient), whereas during (**b**) the trans-tracheostomal protocol, the patient exhales (i.e., negative airflow exiting the mouth of the patient). (**c**) CAD view of the extra-thoracic airway model with anatomic regions highlighted by color. g = Gravity; T-O = Trans-oral; T-T = Trans-tracheostomal.

We recall that the present geometry is based on the upper part of the Lovelace Respiratory Research Institutes A model [24]. The anterior oral cavity was created from in vivo dental impressions of a living Caucasian male at approximately 50 percent of the full opening. To investigate the effect of synchronizing the aerosol puff release time with the quiet breathing pattern under realistic inhaling conditions, we used the breathing profile shown in Figure 3a,b that models an average human adult, where complete inhalation and exhalation are expected to last 2.14 s each, with a tidal volume (TV) of 0.5 L and a peak inspiratory flow rate of 22 L/min [25].

### 2.4. Mesh Generation

The airway geometry was segmented in Meshmixer (Autodesk, San Rafael, CA, USA) for different sections and then discretized using tetrahedral elements in ICEM (ANSYS Inc., Canonsburg, PA, USA), with prism layers at the airway walls. The resulting mesh was imported into Fluent (ANSYS Inc., Canonsburg, PA, USA) and converted into a polyhedral mesh, the benefits of which for simulating respiratory flows have been recently discussed [26]. Rigorous mesh convergence tests were first performed (i.e., ranging from 2M to 8M tetrahedral cells) to eventually select a final mesh of ~4M cells, with up to 3 prism layers for near-wall refinements. Refinements based on curvature were also included to deliver a high-quality mesh. The larynx is the narrowest part of the upper airway, and it generates higher velocities and turbulence. Therefore, as a standard approach in numerical simulations, the velocity magnitude at a section in the larynx was compared with the finest mesh size to ensure that the maximum variations in the selected mesh were <1% of the corresponding values.

### 2.5. Airflow and Aerosol Transport Simulations

The flow of the continuous phase (i.e., air, ρ_f_ = 1.225 kg/m^3^ and µ_f_ = 1.7894 × 10^−5^ kg/(m-s)) is governed by the mass and momentum conservation equations (i.e., Navier–Stokes equations), which are numerically solved using the finite volume method (FVM) that can be solved in a coupled or segregated manner using, here, a commercial solver (i.e., Fluent 19.3, ANSYS, Inc.). Owing to the high flow rates during a breathing maneuver, in conjunction with the intricate shapes of the extra-thoracic model, the flow typically changes from laminar to turbulent regimes, physically characterized by the presence of a wide range of time and length scales in the flow field. For such flow scenarios, the k − ω − SST (Shear Stress Transport) model is comparatively accurate in simulating the relevant airflows in the respiratory tract [27,28,29], also taking into account low-Reynolds corrections.

A second-order implicit scheme is used for the transient formulation, with a time step of 10^−3^ s to ensure good accuracy in resolving the unsteady flows throughout the relevant inhalation cycle. To discretize the diffusion terms, a central difference technique is applied. To decrease numerical diffusion in the unstructured three-dimensional (3D) mesh, a second-order upwind approach is utilized to discretize the advection terms. A second-order implicit temporal discretization was applied for the unsteady (i.e., time-dependent) simulations. A segregated solver is then used to solve the resultant system of equations. The SIMPLE algorithm is used to solve the governing equations by coupling velocity and pressure. The volumetric flow rate at the mouth inlet (see Figure 2a,b) is defined in terms of the time-varying velocity inlet boundary condition, and the no-slip boundary condition is assigned on all the walls of the geometry. The outlets are prescribed with the outflow boundary condition, with a weighted flow rate defining the realistic mass flow distribution previously used by Lizal et al. [22] in their in vitro experiments for the same model.

The modeling of particle transport is directly coupled with the modeling of the airflow. As the airflow is solved using a Eulerian framework, particle motion can be described by either a Eulerian or Lagrangian approach. As the particle-laden airflow is dilute (i.e., the volume fraction of particles is typically <0.1%), one-way coupling between the air and particle flow fields may be employed for numerical simulations. We used the Lagrangian-based Discrete Phase Model (DPM), which treats air as a continuum phase and particles as a dispersed phase. A set of inert, spherical, micron-sized particles (1–12 μm) with a particle density equal to 1230 kg/m^3^ were uniformly injected at a peak inspiratory flow rate from the mouth inlet surface in one case (i.e., trans-oral) and from the cannula inlet surface in the other (i.e., trans-tracheostomal). This was performed by considering a mass flow rate equal to 10^−6^ kg/s for each size, with an initial velocity equal to 100 m/s from a pMDI device [30,31].

Neglecting electrostatic effects and hygroscopic growth, we modeled only viscous drag and gravitational sedimentation forces. As the size of the particles considered in the present study is greater than 1 µm, the effect of the Brownian force on particle deposition is largely negligible and may be neglected [25]. Any given particle is considered to be deposited in the model once it comes into contact with a wall, defined in Fluent with trap boundary conditions on the walls. Deposited particles were categorized by region (Figure 3c): (i) oral cavity, oropharynx and hypopharynx; (ii) supraglottic larynx; (iii) subglottic larynx; (iv) tracheostomy tube; and (v) trachea. The glottis itself was omitted because its surface area is relatively negligible and irrelevant to the study hypothesis. Resulting deposition fractions (DFs) were extracted based on the relative mass of deposited particles in each respective region.

## 3. Results

### 3.1. In Silico Aerosol Deposition

**Figure 4:**
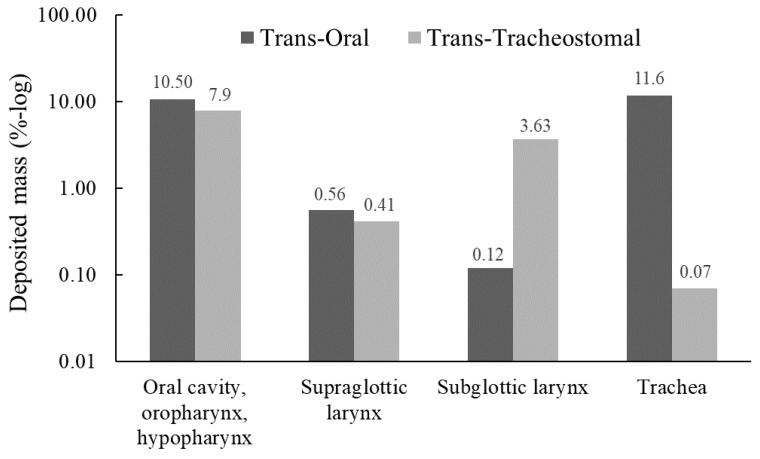
Distribution (logarithmic scale) of deposition fractions (DF) across the different regions (as defined in Figure 3c) for the trans-oral and trans-tracheal techniques.

Figure 4 presents the deposited mass fraction (DF) of inhaled particles in the extra-thoracic model according to the trans-oral and the trans-tracheostomal techniques. Briefly, for the trans-oral inhalation, 66.42% of the particles reached the lower airways and lungs, leaving 33.57% deposited within the upper airway segment. Conversely, for the trans-tracheostomal retrograde technique, 85.09% of the particles exited through the mouth, leaving 14.90% deposited within the model. The highest deposition fractions in the trans-oral technique simulation were in the trachea (11.60%), followed by the oral cavity, oropharynx, hypopharynx (10.5%) and the supraglottis regions (0.55%). Only 0.11% deposited in the subglottis. The highest deposition fractions in the trans-tracheostomal retrograde simulation were in the oral cavity, oropharynx and hypopharynx (7.90%), followed by the subglottis (3.63%) and the supraglottis (0.40%) regions. The deposition fraction in the subglottis was 33 times higher (3.63%) in the retrograde trans-tracheal technique compared to the trans-oral technique (0.11%).

**Figure 5:**
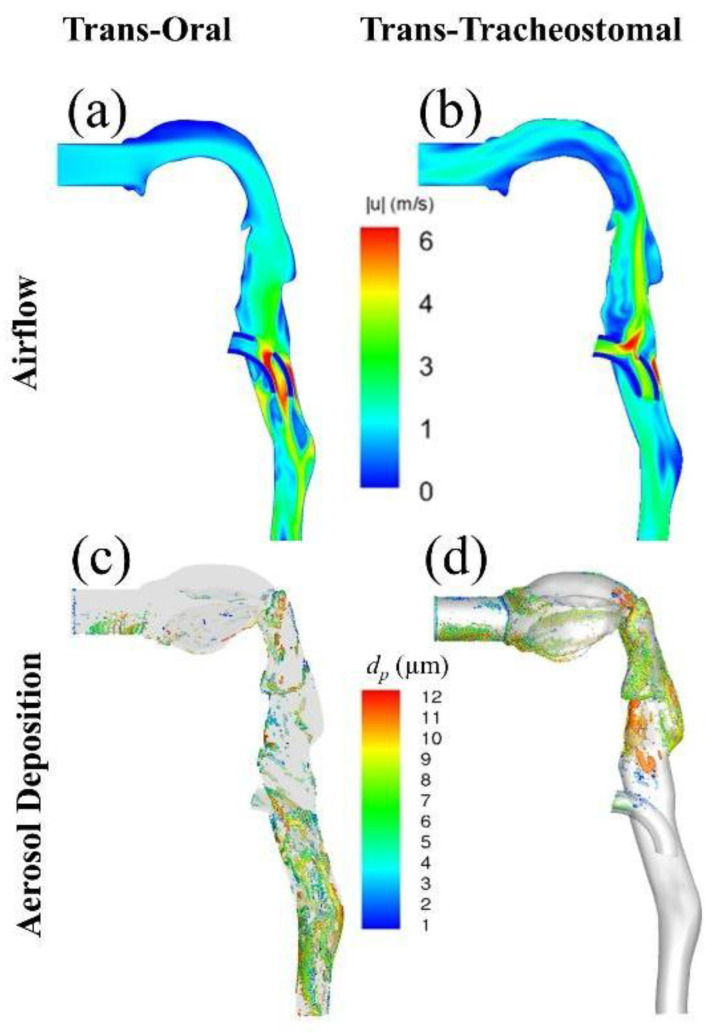
Comparison of instantaneous velocity magnitudes at sagittal plane during (**a**) peak inhalation in the trans-oral model and (**b**) peak exhalation in the retrograde trans-tracheostomal model. Comparison of aerosol deposition between (**c**) trans-oral and (**d**) trans-tracheostomal models.

Figure 5 presents a graphic comparison of airflow and aerosol deposition patterns between the trans-oral and trans-tracheostomal techniques.

### 3.2. Patient Cases

In the following section, we describe the clinical course of four patients following airway surgery and treatment with the TRCI technique.

1. Case 1

A 60-year-old man presented with severe progressive dyspnea 2 months following a week of endotracheal intubation due to a complicated coronary artery bypass graft surgery. In his presentation to the emergency room, he had notable stridor with deteriorating vital signs. Two attempts to perform endotracheal intubation failed, and therefore, he underwent an emergency tracheostomy. Following a short recovery, the patient was breathing spontaneously via tracheostomy. Laryngeal examination showed findings of severe circumferential subglottic stenosis (grade III Cotton Myers stenosis).

During the next six months, the patient underwent two interventions: the first included CO2 laser radial incisions in the stenotic subglottic area with subsequent balloon dilation, which brought mild and only temporary improvement in speech and breathing. The second intervention included a posterior subglottic granulation tissue resection, a submucosal resection of the first tracheal ring protruding into the lumen, and Co2 laser radial incisions with subsequent balloon dilation. Last, the patient was inserted with a 14 mm-diameter stent in the subglottis.

**Figure 6:**
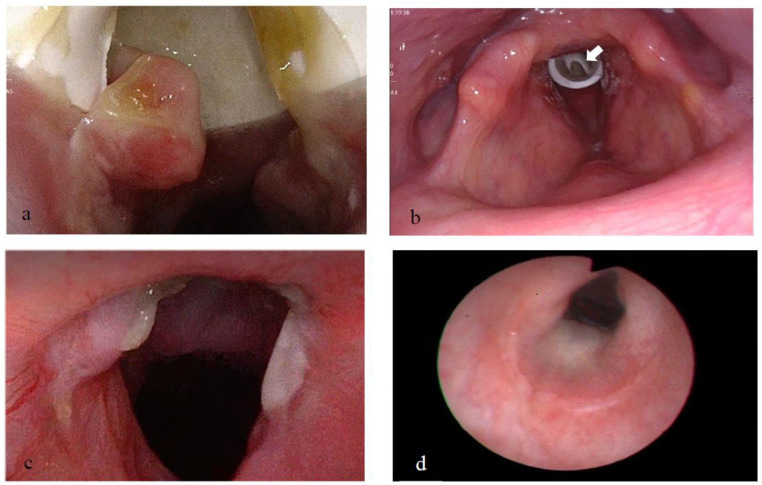
(**a**) Two months following interventions, under TRCI treatment: mild granulation tissue in the subglottis just below the subglottic stent. (**b**) Three months following interventions, under TRCI treatment: cannula fenestration is clearly seen through the stent (arrow). (**c**,**d**) Following stent removal and decannulation: grade 1 Cotton Myers subglottic stenosis. (**c**) Superior view; (**d**) Inferior view. TRCI = Trans-tracheostomal Retrograde Corticosteroids Inhalation.

Treatment with TRCI was initiated following the procedure, according to protocol. During a 4-month follow up period, laryngeal examination showed only mild granulation tissue growth in the subglottic and supra-stomal area, above and below the stent, respectively (Figure 6a,b). The stent was then removed, and the patient continued TRCI treatment for another two months. Finally, laryngeal examination showed only a mild subglottic stenosis (grade 1 Cotton Myers) before he was successfully decannulated (Figure 6c,d).

2. Case 2

A 58-year-old woman presented with progressive stridor and hoarseness, 3 months following a 10-day period of intubation and ventilation due to acute myocardial infarction. She underwent tracheostomy, and direct laryngoscopy revealed severe, multilevel glottic, subglottic and tracheal stenosis. In the following two years, she underwent multiple interventions, including posterior cricoid split with placement of costal graft and three balloon dilations. Following these interventions, major improvement was seen in the glottic and subglottic areas. However, the suprastomal area was still concentrically stenotic. Further granulation tissue was removed in the suprastomal and subglottic area, and a stent was inserted.

**Figure 7:**
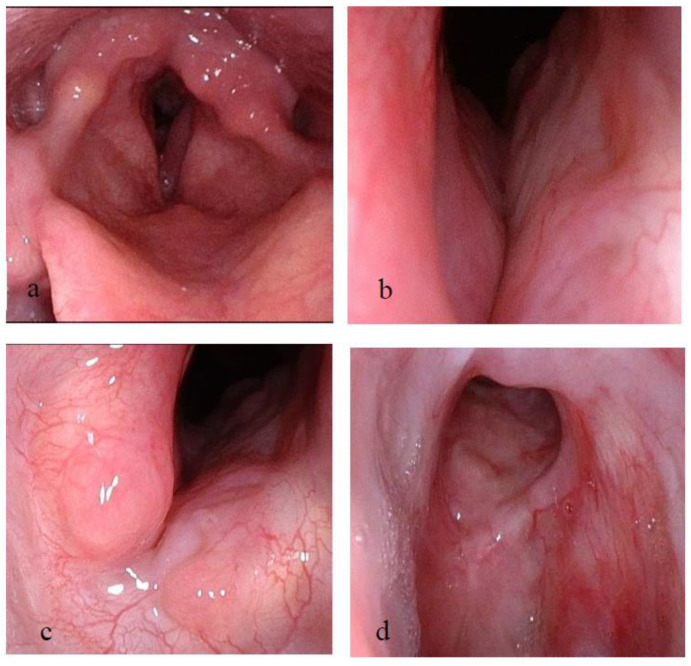
(**a**,**b**) Laryngeal examination immediately following stent removal showing resolution of the suprastomal stenosis and a residual grade 2 Cotton Myers subglottic stenosis. (**a**) Superior view; (**b**) Inferior view. (**c**,**d**) Eighteen months following stent removal, under regular TRCI treatment, no restenosis and no granulation is seen in laryngeal examination (inferior view). TRCI = Trans-tracheostomal Retrograde Corticosteroids Inhalation.

The patient then started TRCI, according to protocol. During a 4-month follow-up period, an improvement in voice production was noticed, and the patient was able to breathe with a blocked cannula for short periods of time. The stent was then removed, with a residual grade 2 Cotton Myers subglottic stenosis (Figure 7a,b). Under the TRCI protocol, no granulation was noticed. The patient also had morbid obesity and high-grade chronic obstructive pulmonary disease. Thus, decannulation was not attempted. The patient is currently 18 months following stent removal and using TRCI treatment regularly. Periodic laryngeal examinations show no deterioration in laryngeal findings (Figure 7c,d).

3. Case 3

**Figure 8:**
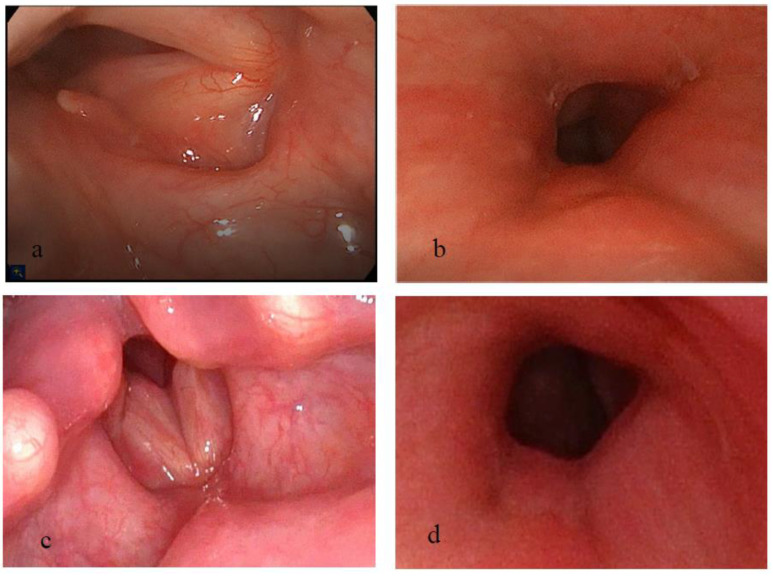
(**a**,**b**) Laryngeal examination immediately following right submucosal arytenoidectomy. (**a**) Superior view; (**b**) Inferior view. (**c**,**d**) After bilateral submucosal arytenoidectomy, 10 months following the initiation of TRCI treatment, no deterioration is seen in laryngeal examination. (**c**) Superior view; (**d**) Inferior view. TRCI = Trans-tracheostomal Retrograde Corticosteroids Inhalation.

A 58-year-old woman presented with progressive dyspnea 6 months following a period of 1 month that she was ventilated via an endotracheal tube and had an extracorporeal membrane oxygenation, due to severe decompensated heart failure exacerbation. Laryngeal examination revealed glottic and subglottic stenosis, and she therefore underwent immediate tracheostomy. In the following year, she underwent four procedures: the first two procedures included glottic and subglottic balloon dilations, and the next two procedures included submucosal arytenoidectomy and lateralization, first on the right and then on the left side. Between these two procedures, the patient started to use TRCI, according to protocol. A significant improvement in her airway was noticed following these interventions, with only a residual grade 1 Cotton Myers subglottic stenosis (Figure 8a,b). The patient continued TRCI for 10 months, with no noticeable deterioration on laryngeal examinations (Figure 8c,d). The tracheostomy tube was gradually narrowed until the patient was successfully decannulated.

4. Case 4

**Figure 9:**
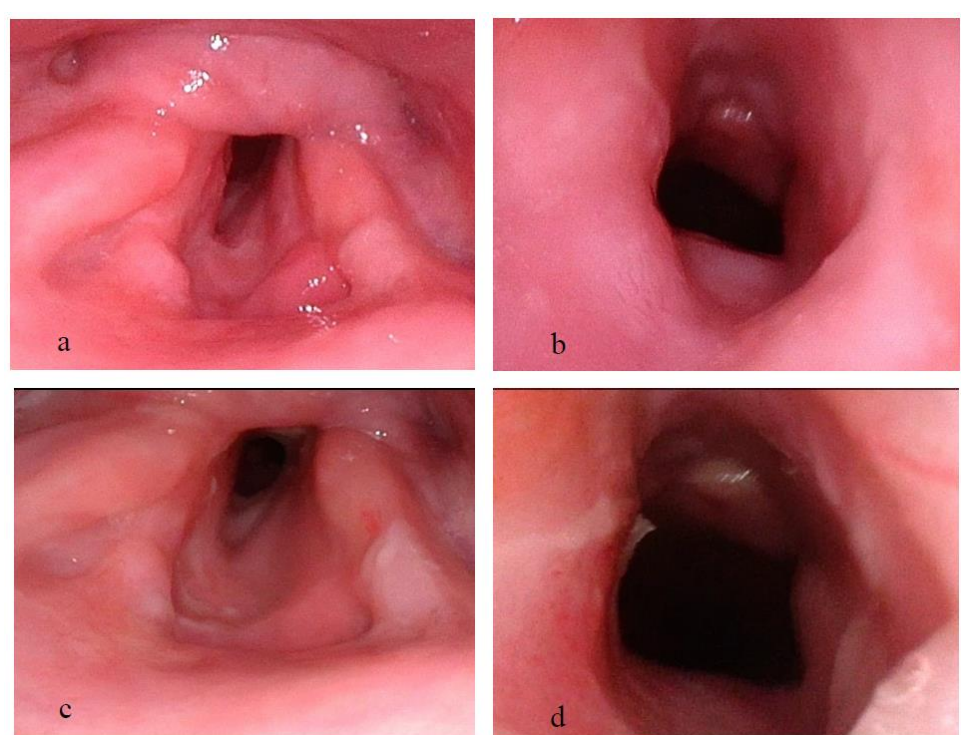
(**a**,**b**) Laryngeal examination immediately following stent removal showing mild granulation tissue in the supraglottic area and a residual grade 1 Cotton Myers subglottic stenosis. (**a**) Superior view; (**b**) Inferior view. (**c**,**d**) Ten months following the initiation of TRCI treatment, no deterioration is seen in laryngeal examination. (**c**) Superior view; (**d**) Inferior view. TRCI = Trans-tracheostomal Retrograde Corticosteroids Inhalation.

A tracheostomy-dependent 49-year-old woman presented with notable aphonia. In the previous three years, she underwent five endoscopic airway procedures after she suffered the adverse effects of radiation therapy due to laryngeal high-grade dysplasia. Laryngeal examination at her presentation revealed severe multilevel stenosis including the glottic, subglottic and tracheal regions. The first surgical intervention included balloon dilation of the stenotic glottic, subglottic and tracheal regions, followed by insertion of a 14-gauge stent to the glottic and subglottic regions above the tracheostomy tube. The stent was removed 4 months later, with major improvement seen in all previously narrowed regions, and the patient was able to breath with a blocked tracheostomy tube, smell and speak. However, in the next 6 months, the patient reported relapse, and examination showed severe glottic and subglottic stenosis. The surgical procedure was repeated. This time, the stent was removed one year following its insertion and revealed major improvement in the glottic and subglottic area and only mild granulation tissue in the supraglottic area (Figure 9a,b). The patient then started TRCI treatment, according to protocol, for 10 months. During her follow-up, the patient was able to spend most of the day with a blocked tracheostomy tube and speak. Laryngeal examination 10 months following stent removal showed a satisfying glottic opening and grade 1 Cotton Myers subglottic stenosis (Figure 9c,d). The tracheostomy tube was gradually narrowed until the patient was successfully decannulated.

The main characteristics of the four patients are summarized in Table 1.

## 4. Discussion

This study presented a novel inhalation technique in tracheostomized patients, designed to maintain surgical results and prevent granulation tissue regrowth and subsequent restenosis. The rationale for TRCI is the attempt to deliver a much higher concentration of corticosteroids to the suprastomal and subglottic areas while minimizing systemic spread to the lungs. This is achieved by forced exhalation during the activation of the inhaler and forcing the drug to exit through the fenestration in the tracheostomy tube upwards, towards the larynx, pharynx and oral cavity.

Our study showed an advantage of trans-tracheostomal inhalation over trans-oral inhalation in the amount of drug particles that deposit in the subglottis (total deposition fraction of 3.63% for trans-tracheostomal vs. 0.11% for trans-oral, ~30 times higher). The low deposition fraction of drug in the subglottis via trans-oral inhalation was also observed in recent studies by Frank-Ito et al., which showed drug depositions between 0.06% to 2.6% in models of subglottic and tracheal stenosis [19,32]. These low fractions emphasize the need to increase drug deposition in the stenotic areas. Suggested options in previous studies included the use of a spacer chamber and mild actuation of the inhaler, along with tidal volume modification that improved deposition [19,32,33,34]. We believe trans-oral inhalation is relatively ineffective in treating subglottic stenosis, especially in tracheotomized patients who breathe through a tracheostomy tube. Since a considerable number of patients with subglottic stenosis breathe via tracheostomy, the TRCI technique takes advantage of the proximity of the tracheostomy tube to the subglottis and allows for extensive delivery of the drug directly onto the subglottis, via the fenestration in the tube.

Consistent with previous in vitro MDI simulations [23,33,34,35], our study showed that about two-thirds of the drug inhaled trans-orally reached the lower airways and lungs. An advantage of the TRCI technique in that manner is that most particles leave the body through the mouth. This also provides feedback and confirmation for proper performance. This feedback does not exist in the trans-oral technique and is a well-known problem [36]. Second, it provides significantly less unnecessary systemic drug absorption and allows for increased dosage to the stenotic segment. In our study, 85% of the drug inhaled trans-tracheostomally exited the body through the mouth. This also emphasizes that TRCI is appropriate only if the site of stenosis is above the tracheostomy and should not be used for tracheal stenosis below the tracheostomy level. Some patients may experience multilevel stenosis [37]. In such a case, the patient may benefit from both trans-oral and trans-tracheostomal inhalations. This idea was supported by a simulation study of trans-oral inhalation in two multilevel stenosis subjects [32], with a superior deposition shown at the lower (tracheal) stenosis in both subjects. In addition to the lower deposition rate in the lungs, TRCI also demonstrated a lower deposition rate in the oral cavity and pharynx compared to the trans-oral technique (7.9% vs. 10.5%, respectively). This is important because these subsites are known to be susceptible to candidiasis with long-term, high-dose inhaled corticosteroids, making it another potential advantage of TRCI.

Expectedly, our results illustrate a graphic correlation between airflow and aerosol deposition. This warrants that a higher mass of drug will head in the direction of the airflow, which is mostly determined by inhalation or exhalation. The trans-tracheostomal technique uses exhalation and the tracheostomy tube, which allows lower entry to the trachea, to achieve higher subglottic drug deposition. This principle can be used to augment drug deposition in other anatomic locations, for example, in tracheostomized patients by setting a fenestration in the cannula in front of a desired location or locations. It also breaks a paradigm that aerosolized medication (usually called inhalation) must be administered during inhalation of air.

The patient cases presented with various manifestations of multilevel laryngotracheal stenosis and were mostly females (3/4) in the age range of middle to older age (67 ± 6.5), as seen in large cohorts in the literature [3,5,38]. All cases in our series underwent several endoscopic interventions before TRCI was initiated. The etiology for laryngotracheal stenosis was prolonged intubation in 75% of cases (cases 1–3), and Case 4 in this study suffered irradiation-induced stenosis. Overall, the 4 cases in this cohort underwent 19 procedures: 18 endoscopic and 1 open procedure. A systematic review showed that up to 54% of patients presenting with laryngotracheal stenosis will require additional surgery, depending on the etiology and surgery, with pooled recurrence rates of 35% and 54% for iatrogenic (intubation/tracheostomy) and traumatic (including radiation induced) causes, respectively. The systematic review also showed pooled recurrence rates of 44% for endoscopic surgery [39]. In another study, 92 adults with subglottic stenosis underwent 247 endoscopic procedures in a median follow-up of 2.4 years, and another 8 total (9%) patients went on to require an open procedure [5]. Multilevel stenosis, as seen in 3/4 of our cohort, requires more surgeries than other presentations [38]. Thus, the cases in our cohort can be considered within the severe range of presentation and response to treatment.

In this limited cohort, no deterioration in laryngeal examination was seen during a mean period of 10.5 months of TRCI treatment in all patients, and no further surgery was required during treatment. Further, 3/4 of the cohort were successfully decannulated following this treatment. One patient was not decannulated, mainly due to her high-grade chronic obstructive pulmonary disease with frequent exacerbations, but was able to withstand a considerable amount of time with a blocked cannula. A systematic review of cohorts with different presentations of laryngotracheal stenosis showed decannulation rates of 63% with an endoscopic procedure and up to 89% with open procedures. In a recent study with a cohort of 38 patients with multilevel stenosis, only 21.2% were successfully decannulated following a mean of 4 ± 3.9 surgeries, and the decannulation percentage was lower than other focal etiologies [38]. Since most cases in our study (75%) presented at first with multilevel stenosis, decannulation rates in this limited cohort were comparatively high. We believe that TRCI may have an added value in preserving the surgical outcome and preventing the recurrence of granulations and stenosis. This should be further investigated in larger prospective studies

Airway stents were placed and stayed in the airway postoperatively for 4 and 10 months in cases number 2 and 4, respectively. Granulation tissue growth at the free edges of airway stents is a well-known complication and can reach a prevalence of up to 65% [40,41,42]. However, Cases 2 and 4 were treated with TRCI during this period, and only slight granular changes were noticed at the stent edges before it was removed. Therefore, it is likely that TRCI treatment may help in preventing this unwanted outcome. Furthermore, Case 2 suffered extensive granular tissue growth at the edges of the stent when she was previously not treated with TRCI. This difference in outcomes in the same patient, with and without the treatment, supports the hypothesis of the possible role of TRCI in preventing restenosis.

An important limitation of this study was that the model depicts a normal non-stenotic airway. We hypothesize that conducting the same simulation in a model of subglottic stenosis would result in higher subglottic deposition via the TRCI technique. This assumption is based on the proximity of the subglottis to the tracheostomy tube fenestration, where the particles leave the cannula and are launched upwards. Thus, a stenotic subglottis would more likely block a higher number of particles. Our study depicts the simulation of a single inhalation pressure with a narrow range of particle sizes and does not examine the effect of a spacer. Further, substantial variability exists between one patient’s airway and another’s; this, along with the different possible locations of the tracheostomy, different tube sizes and different locations of stenosis, highlight the case-specific conclusion of this study and emphasize the need for further research. Notably, this study has a very limited cohort and no control group. Due to these reasons and the diversity in presentations and prior surgical treatments of this study’s cohort, no individual factor, including TRCI, can be isolated as the reason for the maintenance of postoperative results and decannulation success. While our treatment protocol utilized fluticasone propionate, we acknowledge that other inhaled corticosteroids may affect the efficacy of our method and require further study. Nonetheless, the same considerations for drug selection as in chronic obstructive pulmonary disease and asthma, such as drug potency, receptor selectivity and safety profile, may apply. Furthermore, the trans-tracheostomal retrograde technique has the potential to be used with various inhaled drugs for different indications.

## 5. Conclusions

This study presents a trans-tracheostomal, retrograde, corticosteroid inhalation technique, which may raise the subglottic deposition rate, while avoiding superfluous aerosol deposition in the broader respiratory tract. Initial clinical experience with this technique shows encouraging results in patient cases. This preliminary study represents a promising proof-of-concept, and further in vitro and clinical studies are needed to conclude on its clinical efficacy.

## Figures and Tables

**Table 1:** Summary of the case series characteristics.

Case Num.	1	2	3	4
Age	60	58	58	49
Sex	M	F	F	F
Clinical presentation	Dyspnea, stridor	Dyspnea, stridor aphonia	Dyspnea, stridor	Dyspnea, aphonia
Etiology of stenosis	Prolonged intubation and tracheostomy	Prolonged intubation and tracheostomy	Prolonged intubation and tracheostomy	Irradiation induced stenosis
Laryngeal Findings at presentation	Subglottic stenosis	Bilateral vocal fold immobility, multilevel glottic, subglottic and tracheal stenosis	Posterior glottic stenosis and subglottic stenosis	Glottic, subglottic and tracheal stenosis
Cotton Myers grade at presentation	3	3	2	3
Surgical Interventions	Co2 laser radial incisions, balloon dilation, granulation tissue resection, stent insertion	Posterior cricoid split with placement of costal graft, balloon dilation, stent insertion	Balloon dilation, submucosal arytenoidectomy and lateralization stent insertion	Balloon dilation, granulation tissue resection, stent insertion
Cotton Myers grade following interventions	1	1	1	1
TRCI treatment length (months)	6	16	10	10
Cotton Myers grade following TRCI treatment	1	1	1	1
Decannulation	Y	N	Y	Y

F = female; M = male; TRCI = Trans-tracheostomal Retrograde Corticosteroids Inhalation; Y = yes; N = no.

## Supplementary Materials

The following supporting information can be downloaded at: https://www.mdpi.com/article/10.3390/pharmaceutics15030903/s1, Video S1: The assembly of the trans-tracheostomal retrograde device; Video S2: Trans-tracheostomal Retrograde Corticosteroid Inhalation Technique.
